# Effects of sex and gonadectomy on social investigation and social recognition in mice

**DOI:** 10.1186/s12868-015-0221-z

**Published:** 2015-11-25

**Authors:** Sara A. Karlsson, Kaltrina Haziri, Evelyn Hansson, Petronella Kettunen, Lars Westberg

**Affiliations:** Department of Pharmacology, Institute of Neuroscience and Physiology, Sahlgrenska Academy, University of Gothenburg, Gothenburg, Sweden; Department of Psychiatry and Neurochemistry, Institute of Neuroscience and Physiology, Sahlgrenska Academy, University of Gothenburg, Gothenburg, Sweden

**Keywords:** Social behaviour, Memory, Three-chambered apparatus test, Sex differences, Estrogens, Androgens

## Abstract

**Background:**

An individual’s ability to recognise and pay attention to others is crucial in order to behave appropriately in various social situations. Studies in humans have shown a sex bias in sociability as well as social memory, indicating that females have better face memory and gaze more at the eyes of others, but information about the factors that underpin these differences is sparse. Our aim was therefore to investigate if sociability and social recognition differ between female and male mice, and if so, to what extent gonadal hormones may be involved. Intact and gonadectomised male and female mice were assessed for sociability and social recognition using the three-chambered sociability paradigm, as well as the social discrimination test. Furthermore, we conducted a novel object recognition test, a locomotor activity test and an odour habituation/dishabituation test.

**Results:**

The present study showed that the ability to recognise other individuals is intact in males with and without gonads, as well as in intact females, whereas it is hampered in gonadectomised females. Additionally, intact male mice displayed more persistent investigatory behaviour compared to the other groups, although the intact females showed elevated basal locomotor activity. In addition, all groups had intact object memory and habituated to odours.

**Conclusions:**

Our results suggest that intact male mice investigate conspecifics more than females do, and these differences seem to depend upon circulating hormones released from the testis. As these results seem to contrast what is known from human studies, they should be taken into consideration when using the three-chambered apparatus, and similar paradigms as animal models of social deficits in e.g. autism. Other behavioural tests, and animal models, may be more suitable for translational studies between patients and experimental animals.

## Background

The ability to focus on and recognise other individuals, often referred to as social preference and social recognition, respectively, are prerequisites for individuals to behave adequately in social contexts. Human studies indicate that women perform better than men in tasks measuring face memory [1] and emotion recognition [2]. Women also gaze more at the eyes of others [3] and the risk for autism spectrum disorders is lower in girls than in boys. Hence, sex differences in social preference and social memory are rather established in humans, but the underlying causative factors for these differences are unknown. Interestingly, variations in testosterone levels have been suggested to partially explain some of these differences [4]. In recent studies, testosterone treatment decreased trust and cognitive empathy [5], but also modulated the neural responses to emotional faces [6] and to crying infants in women [7].

In rodents and other vertebrates, it is well-established that testosterone is crucial for sexual dimorphisms in social behaviours, such as aggression, mating behaviours and parental behaviours. The effects of testosterone are mediated by androgen receptors, and after aromatization to 17-beta-estradiol, by estrogen receptors. Male rodents lacking gonads, androgen receptors or estrogen receptors display substantially decreased aggression and sexual behaviours [8–11]. Some studies have shown sex differences in the duration of social investigation [12–14], and testosterone has been suggested to be involved [15–17]. The importance for sex and gonadal hormones has, so far, not been evaluated in the three-chambered apparatus test measuring sociability and social investigation. This is relevant since the three-chambered apparatus test is often used when aiming to understand social deficits seen in autism spectrum disorders, a group of disorders with higher prevalence in males. Furthermore, although estrogens are known to improve social recognition in mice through estrogen receptors [18, 19], and some studies have shown effects of testosterone on long-term social recognition in rats [20, 21], it is not clarified to what extent testis hormones modulate social discrimination in male mice and if social recognition is sexually dimorphic in mice.

As described above, there are inconsistencies and gaps in the knowledge about the role of sex and testosterone for social recognition and social preference. Therefore, the aim of this study was to elucidate how sex and gonadal hormones modulate sociability and social recognition in mice, using the three-chambered apparatus test and the social discrimination paradigm. We initially validated the social discrimination test in male mice, and investigated if social recognition ability was dependent on the gonadal status of the female stimulus mice. Furthermore, sociability and social recognition were investigated in male and female mice, with or without gonads. In order to evaluate if differences in social tests may be caused by related functions, object recognition memory, olfaction and locomotion was also tested on the same mice.

## Methods

### Animals

#### *C57Bl/6N* wild type mice

The mice used in the different experiments (A–D) are described in Table 1. The same set of in-house bred adult *C57Bl/6N* male mice was used in the tests validating the social discrimination paradigm (experiments A and B in Table 1) and the animals used in experiments C and D (Table 1) was purchased from Charles River (Denmark). The purchased *C57Bl/6*N mice in experiments C and D (Table 1) were left to habituate to the animal facility for more than 2 weeks before commencing the study. There was approximately 1 week between experiments A and B and experiments C and D, respectively. In experiments investigating the importance of gonads (D in Table 1), half of the male and female groups were sham-operated, and remaining animals were gonadectomised (GDX) 3 weeks prior to the study (see below). In total, two mice did not survive the surgery procedure. Four groups were generated: intact males n = 14, intact females n = 14, GDX males n = 14 and GDX females n = 15. All mice used in this study were virgins.

**Table 1:** The test and stimulus mice used

	Test	Test mice	Age (months)	N	Source	Stimulus mice	Age (months)	Source
		Gonadal status/sex				Gonadal status/sex		
A	Social recognition	Intact ♂	3–5	28	In-house	GDX ♀	8	Charles river^a^
B	Social recognition	Intact ♂	3–5	28	In-house	Two novel (GDX ♀)	8	Charles river^a^
C	Social recognition	Intact ♂	4–5	20	Charles River^a^	GDX and intact ♀	6	Charles river^a^
D	Social recognition	Intact and GDX ♀ and ♂	5–6	57	Charles River^a^	GDX ♀	7	Charles river^a^
	Three-chambered apparatus			57				
	Object recognition			57				
	Locomotor activity			57				
	Olfactory test			54				

^a^Denmark

All stimulus animals (A–D) were of the *C57Bl/6N* strain (Table 1). Female stimulus mice were GDX shortly after arrival to the animal facility and were used in tests after at least 6 weeks of recovery. All stimulus mice were single-housed 1 week prior to testing in order for them to gain an individual scent. Throughout the social tests, stimulus animals were presented to the test animals in a wire corral (Galaxy pencil cup [22]). The stimulus mice were habituated to the wire corrals during 15 min for 2 days prior to the social tests to avoid unnecessary stress causing disturbing behaviours like aberrant bar biting. Our study aims warrant a relatively neutral social stimulus. Thus, in line with many other previous studies we chose to use GDX females although juvenile mice also are commonly used.

#### Surgery

The gonadoectomy was performed via an abdomen midline incision under anaesthesia with a 3:12 vol/vol mixture of ketamine (Ketalar 10 mg/ml, Pfizer) and xylazine (Rompun Vet 20 mg/ml, Bayer Animal Health). During the surgery ovaries and testicles were removed from the female and male mice, respectively. Animals were allowed to recover in group for 4 weeks before testing. All efforts were made to prevent any suffering of the animals during the surgery.

### Behavioural testing

#### Experimental conditions

One week prior to social experiments, the test animals were habituated to new standard test cages during 10 min for 5 days. The rooms used for the experiments had an illumination of ~20 lux and were spared from strong smells and sounds. The corrals, objects and the three-chambered apparatus were cleaned with 70 % ethanol followed by water, before and between the social tests. All mice were held in a conventional animal facility with a 12 h light/12 h dark cycle (lights on at 6.00 AM) and were given ad libitum access to food and water and the behavioural tests were performed between 9 am and 16 pm. Forty-five minutes before commencing the test, all the mice were transported from the housing room to the testing area, and they were let to acclimatise to their new surroundings, as well as recover from any stress caused by the transportation. All procedures were subjected to approval by the Ethical Committee on Animal Experiments, Gothenburg, Sweden (permit number 313-2011) and performed accordingly.

#### Social discrimination test

The social discrimination test, developed by Macbeth et al. [23], was used to test the social memory, i.e. the ability to remember an already encountered conspecific. The test consisted of two collection parts: the social investigation part (sample) and social recognition part (choice) with a 30 min inter-trial-interval. *Social investigation:* The focal animal, i.e. the specific mouse tested, was placed in a transparent test cage (41 × 25 × 14 cm) containing bedding, and was then allowed to habituate for 15 min. After the initial habituation period, two corrals were placed in the cage and the mouse was habituated to the wire corrals for approximately 30 min. Following this, one of the wire corrals was removed from the cage and a stimulus mouse was placed in the remaining corral. When the stimulus mouse was introduced to the focal individual, the sampling sessions were recorded with an overhead video camera. In order to secure an at least as robust social recognition in the actual experiments (C and D) as seen in the validation experiments (A and B) the sampling time was increased from the 5 min used in A and B to 10 min in C and D. After the first experimental session, focal mice were left in the test cage with the two empty corrals for 30 min. *Social recognition:* The testing of short-term memory commenced when the focal mouse was presented with the familiar stimulus mouse from the sample session, and a novel mouse. They were both introduced at the same time and were enclosed in two separate corrals respectively. The social memory score was calculated the following way: time exploring novel mouse/(time exploring novel mouse + familiar mouse), with a ratio above 0.5 indicating an intact social memory.

#### Social investigation in the three-chambered social approach test

Sociability was tested with the three-chambered apparatus. This test was developed by Moy et al., [22] to screen for sociability in mice and measure preference for a novel conspecific vs. an empty corral. Both the duration of time in each chamber and time spent sniffing were recorded. *Sociability test:* After acclimatisation to the test room, focal mice were put into the three-chambered apparatus with two empty corrals to freely explore and habituate to the test arena for 20 min. Following habituation, the mouse was led to the middle chamber and the openings in the apparatus were closed. A stimulus mouse was then placed in a corral in one of the side chambers together with an empty coral in the other side chamber. The doors between chambers were then removed and the sociability test was initiated. Each experimental session lasted 10 min and was recorded with an overhead video camera.

#### Novel object recognition test

The novel object recognition (NOR) task is used to study recognition memory in rodents [24]. In the present study it was applied to verify presence of object memory, as well as to test for general object investigation. NOR was conducted in a similar way as the social discrimination test: The focal mouse was placed in a test cage (41 × 25 × 14 cm) for 30 min habituation, and the test started when two similar objects were presented for 5 min. The novel object memory was assessed using a 30 min interval between the two identical objects, the sample session, and the presentation of the familiar object and a novel object, the choice session. During both the sample session and the choice session, objects were located in opposite and symmetrical corners of the test arena. Localisation of novel objects and familiar objects was counterbalanced and this modification was made to reduce object and place preference effects. One triangular, one cylindrical, one cubic and one round object, each of different materials, were used. Similar to the social recognition score, an object recognition score was calculated: time exploring novel object/(time exploring novel object + familiar object) where a ratio above 0.5 indicates object memory.

#### Locomotor activity/open field test

To screen for normal motor function of focal mice, locomotion was measured using an open field arena that consisted of a glass Pyrex box with the following dimensions: 60 × 60 × 60 cm (Kungsbacka Mät-och reglerteknik, Sweden). Mice were allowed to habituate for 1 h within the test room, and then placed in the test box; lights were turned off and the focal animal was left to explore the arena for 40 min. Photocells were arranged both horizontally and vertically, covering the complete area of the box. Between the experiments, the boxes were cleaned to remove olfactory cues. One intact female was considered as an outlier (>±2 SD from mean) and was excluded from the study.

#### Olfactory habituation/dishabituation test

Since olfactory cues are crucial for social behaviours in mice, we executed an olfactory habituation/dishabituation test [25] assessing the ability to detect and discriminate between different odours. The focal mouse was habituated to a new test cage containing a clean cotton tip for 30 min prior to testing and was then presented to the odours with a 1 min inter-trial interval. The test consisted of a presentation of different odours in a sequence; each odour was subsequently presented on a cotton tip with duration of 2 min × 3 repeats: water, non-social odour number 1, non-social odour number 2, followed by a social odour. The non-social odours were lemon oil (Sigma-Aldrich, Sweden) and cinnamon oil (AROMA Creative AB, Sweden). The social odour was obtained by swabbing the cage of a female mouse with a cotton tip. Data from three animals (two intact male and one GDX females) was lost, due to computer problems during testing, leaving a total of 54 animals.

#### Measurement of circulating testosterone levels

The mice were terminated between 9 am and 1 pm, and blood was collected in a 1.5 ml tube, centrifuged for 10 min at 4500×*g*, and the plasma was pipetted off into clean microcentrifuge tubes. Plasma was stored in −80 °C until it was analysed for testosterone content using an ELISA assay (EIA-5179, DRG Instruments GmBH, Germany). The analysis showed that gonadectomy of the animals was successful in all cases except in one of the GDX males that was consequently excluded from the behavioural analyses (leaving n = 14). In the animals used for experiments the male group had an average testosterone level of 6.1 ± 0.6 ng/ml, females 0.1 ± 0.1 ng/ml, GDX females 0.1 ± 0.1 ng/ml and GDX males 0.07 ± 0.3 ng/ml.

### Scoring criteria

Scoring criteria for the social tests are described in Yang et al. and Macbeth et al. [23]. In brief: Sniffing directed to the stimulus animal or to any part of the mouse (e.g. the tail) positioned outside of the wire corral, as well as insertion of the nose and forepaws between the corral bars, was scored. Sniffing directed to the upper and top part of the wire corral, sniffing of faeces, bar biting and circulating around the corral without sniffing, did not qualify for scoring. In the three-chambered apparatus, time spent in each chamber and numbers of entries into each chamber were also scored. For the novel object recognition and olfactory tests, the scoring criteria were met when the sniffing occurred approximately 2 cm from the objects or cotton swabs. Scoring these behaviours was performed by one trained observer, who was unaware of the status of the mice.

### Statistical analysis

Statistical associations between groups and within groups were estimated using a linear mixed model in the MIXED procedure (PROC MIXED) of SAS 9.3 (SAS Institute, Inc., Cary, NC, USA). PROC MIXED is a repeated measurement analysis that allows both fixed and random effects/variables. This model was preferred since it includes missing (random) values and also uses maximum-likelihood estimation instead of sums of squares. Social and object recognition memory scores were analysed in SPSS using the one-sample *t* test, with a cut-off value of 0.5 (IBM SPSS Statistics for Windows, Version 19.0. IBM Corp., USA). A *p*-value less than 0.05 was considered as statistically significant.

## Results

### Validation of the social discrimination model

**Fig. 1:**
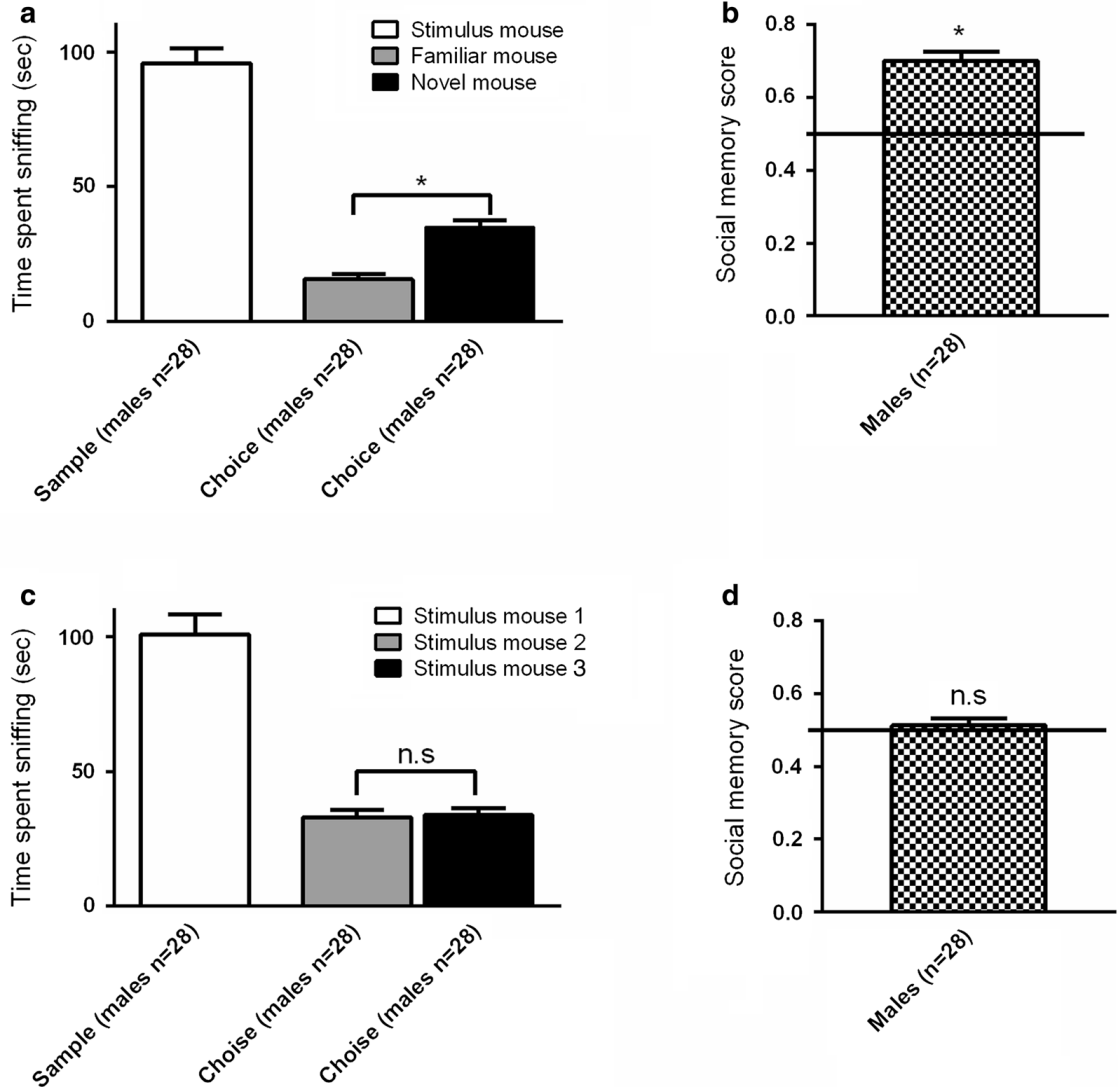
Validation of the social discrimination model measuring social recognition. Male focal mice discriminated between novel and familiar **a**, **b** or only novel GDX female stimulus mice **c**, **d**. **a**, **c** *White bars* represent the amount of time spent sniffing a novel conspecific contained in a wire corral during the 10 min sampling session. *Grey* and *black bars* represent the amount of time spent sniffing different stimulus mice during the 5 min choice session. **b**, **d** *Chequered bars* represent social memory scores. *Error bars* represent mean ± SEM, *p < 0.05

In the choice session of the first experiment (A) the males clearly discriminated between novel and familiar stimulus mice (*p* < 0.0001, Fig. 1a). They also displayed social recognition which was measured using the social memory score (*p* < 0.0001, Fig. 1b). In the choice session of the second experiment (B) focal animals performed as expected, by not discriminating between the two novel conspecifics (*p* = 0.79, Fig. 1c and *p* = 0.56, Fig. 1d). Hence, our results verify the validity of the model.

### Effects of stimulus mice in the social discrimination model

**Fig. 2:**
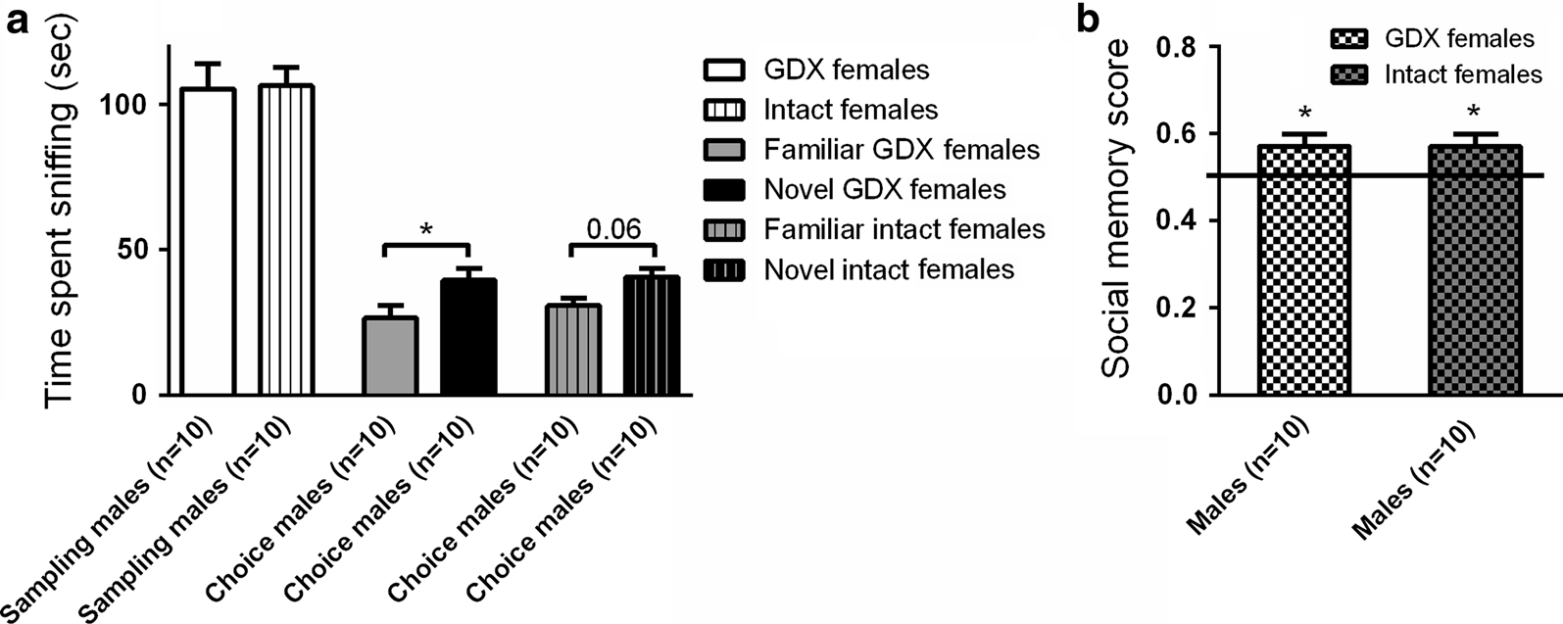
Importance of gonadal status of the stimulus animals for social recognition. **a** Male test mice investigated novel and familiar GDX (*open bars*) or intact (*striped bars*) female stimulus mice in the social discrimination model. *White bars* represent the amount of time spent sniffing a novel conspecific contained in a wire corral during the 10 min sampling session. The *grey* and *black bars* represent the amount of time spent sniffing a novel mouse and a familiar mouse, respectively, during the 5 min choice session. **b** *Chequered bars* represent social memory scores. *Error bars* represent mean ± SEM, *p < 0.05

In the first session of the social discrimination test, where focal mice investigated one novel mouse caged in a wire corral, males spent equal time investigating the GDX animal compared to the time investigating the intact stimulus animal (Fig. 2a). In the second session of the social discrimination test, both male groups, either presented to GDX or to intact stimulus females (Fig. 2a), displayed intact social recognition with respect to social memory scores (within-group comparison *p* < 0.05; Fig. 2a, b).

### Effects of sex and gonadal hormones on social recognition and sociability

**Fig. 3:**
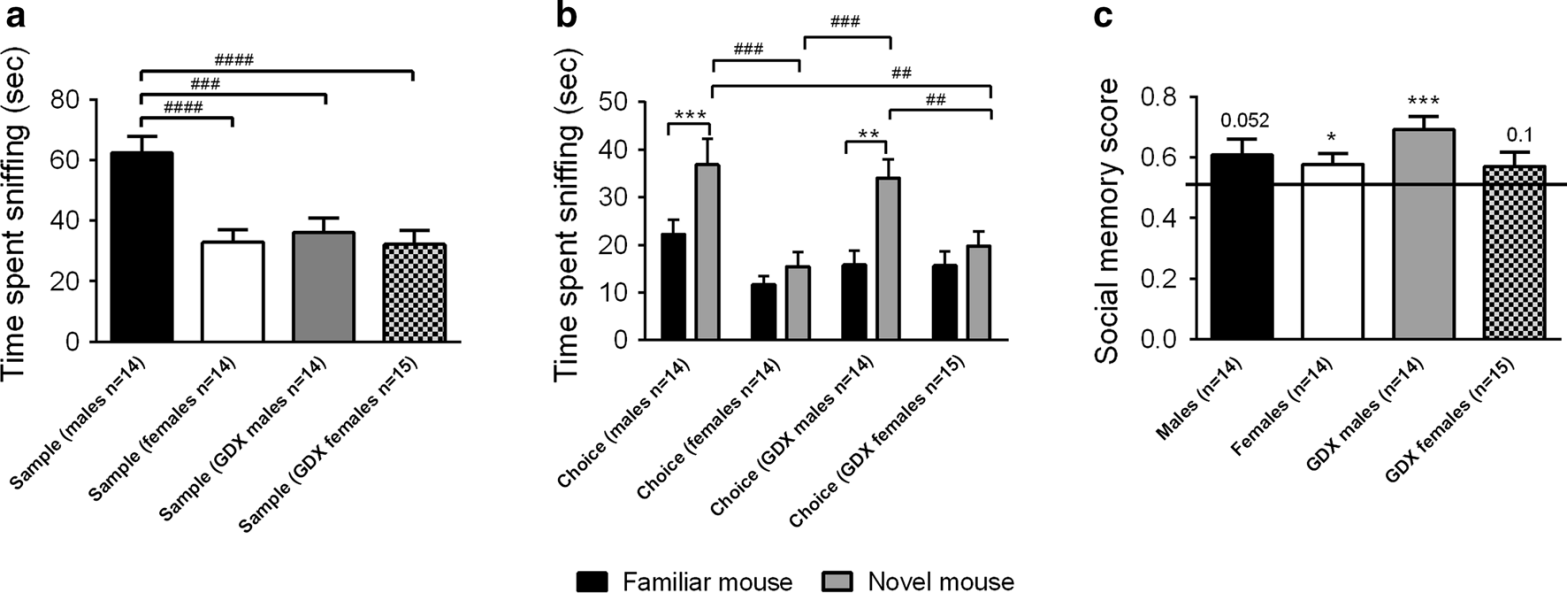
Social recognition measured in the social discrimination model. **a** Amount of time sniffing one mouse contained in a wire corral during the 10 min sampling session. **b** Amount of time spent sniffing a novel mouse or the familiar mouse during the 5 min choice session. **c** Social recognition measured as social memory score calculated from the choice session data. *Error bars* represent mean ± SEM, *p < 0.05, **p < 0.01, ***p < 0.001 (within-group comparison), ^##^ *p* < 0.01, ^###^ *p* < 0.001 and ^####^ *p* < 0.0001 (between-group comparison)

In the first session of the social discrimination test, intact males spent a significantly greater amount of time exploring stimulus animals compared to both female groups and GDX males (between-group comparison *p* < 0.001; Fig. 3a). In the second session measuring social memory, both male groups displayed social recognition when measured as social discrimination (Fig. 3b) and social memory score (Fig. 3c) whereas intact females only did so when using the social memory measure, and GDX females did only show a tendency for significance for any of the measures (Fig. 3b, c). Between-group comparisons showed that the two male groups spent more time investigating the novel mouse than the two female groups (*p* < 0.01; Fig. 3b).

**Fig. 4:**
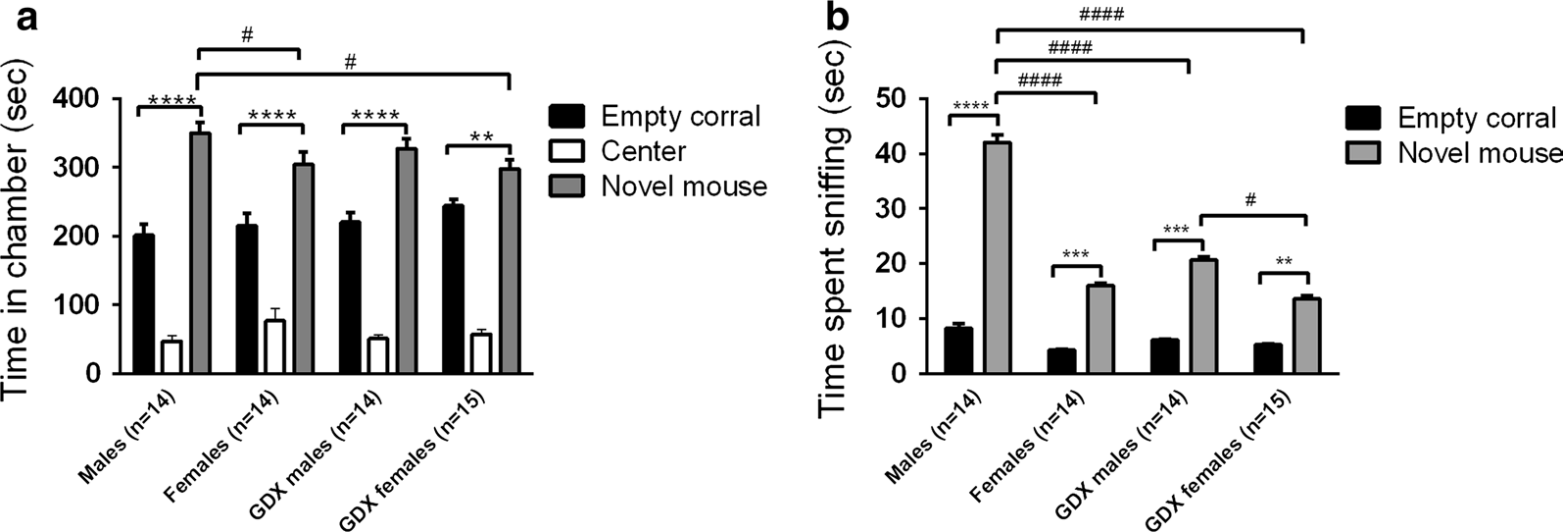
Sociability measured with the three-chambered apparatus test. **a** Amount of time spent in each chamber during the 10 min test of sociability. **b** Amount of time spent sniffing the novel mouse or the empty corral. *Error bars* represent mean ± SEM, **p < 0.01, ***p < 0.001 and ****p < 0.0001 (within-group comparison), ^#^ *p* < 0.05 and ^####^ *p* < 0.0001 (between-group comparison)

In the three-chambered test investigating sociability, all four groups (intact males, intact females, GDX males and GDX females) showed sociability, i.e. they spent more time in the social chamber containing a stimulus mouse than in the empty non-social chamber (within-group comparison *p* < 0.01; Fig. 4a). All groups spent more time sniffing the stimulus mouse compared to sniffing empty corrals (within-group comparison *p* < 0.01; Fig. 4b). The between-group comparison also showed that intact males investigated the stimulus mice for a longer time than the mice from the other groups did (between-group comparison *p* < 0.001; Fig. 4b). When comparing entries between chambers no differences were identified in any of the groups (*p* > 0.05).

### Effects of sex and gonadal hormones on novel object recognition, odour habituation and locomotor activity

**Fig. 5:**
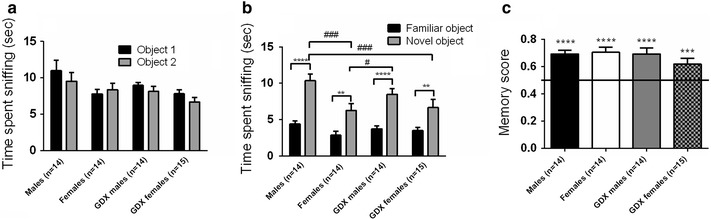
Novel object recognition test. **a** Amount of time spent sniffing two similar objects during 5 min. **b** Amount of time spent sniffing a novel object or a familiar object for 5 min. **c** Object recognition measured as memory score calculated from the choice session data. *Error bars* represent mean ± SEM, **p < 0.01, ***p < 0.001, ****p < 0.0001 (within-group comparison), ^#^ *p* < 0.05 and ^###^ *p* < 0.001 (between-group comparison)

In the first session of the novel object recognition test, where mice investigated two similar objects, all four groups spent equal amount of time investigating the two novel objects showing no preference for side, or other factors (between-group comparison *p* = 0.2; Fig. 5a). In the second session of the novel object recognition test, all groups showed object recognition memory with respect to the object recognition score, measured as time in proximity to the familiar object vs. a novel object (within-group comparison *p* < 0.01; Fig. 5b, c). Between-group comparison showed that intact males spent more time investigating the novel object compared to both female groups (between-group comparison *p* < 0.05; Fig. 5b).

**Fig. 6:**
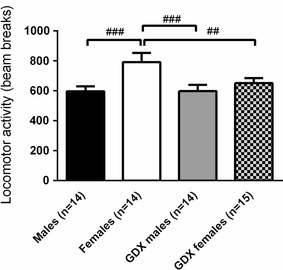
Locomotor activity measured in an open field arena. *Error bars* represent mean ± SEM; ^##^ *p* < 0.01 and ^###^ *p* < 0.001 (between-group comparison)

**Fig. 7:**
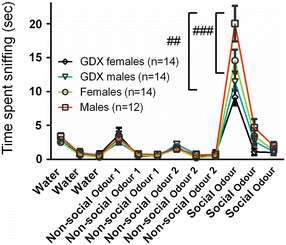
Odour discrimination measured in the odour habituation/dishabituation test. Amount of time spent sniffing water or odour-wet cotton swabs. *Error bars* represent mean ± SEM; ^##^ *p* < 0.01 and ^###^ *p* < 0.001 (between-group comparison)

The locomotor activity test revealed that the intact females showed elevated activity compared to the other groups (between-group comparison *p* < 0.01; Fig. 6). All four groups showed olfactory habituation in the habituation/dishabituation test of olfaction, indicating normal olfaction. Moreover, intact males investigated the social odour significantly longer time than both GDX groups (between-group comparison *p* < 0.01; Fig. 7) and a similar trend was seen when comparing intact males and females (*p* = 0.08).

## Discussion

This study was designed to examine the role of sexual category and the influence of gonadal hormones on social recognition and sociability in mice. Our results clearly show that intact male mice have higher social investigatory behaviour compared to intact females, as well as GDX males and females. Since GDX males showed similar investigation times as intact females and GDX females, the observed difference seems to be testis-dependent. Furthermore, in the novel object recognition test intact males also displayed elevated investigatory behaviour compared to the female groups, suggesting that the sex difference seen in social preference may be partly due to higher level of novelty exploration in intact males than in females. One could also speculate that females are better in encoding social information than males since their investigation time was shorter. Finally, since females displayed elevated locomotor activity compared to males, the sexual dimorphism in exploration of novel conspecifics and objects could not be explained by differences in general locomotor activity.

The testis-dependent difference in social preference was seen in both the three-chambered apparatus test and the social discrimination assay, suggesting that this is a general paradigm-independent sexual dimorphism. This notion is strengthened by the fact that Thor and co-workers reported very similar results in rats investigating pups and pre-pubertal conspecifics in their home cage [15, 16]. Interestingly, the studies by Thor et al. also showed that testosterone treatment of adult female rats fully reversed the sex difference [15, 16], and neonatal androgenisation further increased the sensitivity to exogenous testosterone of female rats as measured by social investigation in adulthood [15, 16]. Also in mice, exogenous testosterone was recently shown to increase social investigatory behaviour [17]. Taken together, these results suggest that the sex difference in sociability is due to the higher levels of testosterone in males compared to females. Moreover, social investigation did not differ between intact and GDX females in the present or in previous studies [15, 16], nor was it increased by estrogen treatment [15, 16]. This indicates that testosterone elevates social investigatory behaviour in females by acting on androgen receptors and not on estrogen receptors after conversion to estradiol.

Although our results are concurrent with those from rats investigating pups [15, 16] as well as same-sex conspecifics [26], it may be speculated—since we used GDX and intact females, as stimuli—that the observed sex difference in social investigation depends on the sex of the stimulus animals and using males as stimulus can further investigate the influence of gender on the stimulus animal. However, previous findings argues against that notion; Ryan and co-workers showed that male *C57BL/6J* mice in the three-chambered apparatus test displayed equal amount of interest in the stimulus animal independent of its sex [27]. In a recent study, elevated social investigation was seen in males when comparing estrogen-treated males and females, exploring either GDX female or male conspecifics. No sex differences were however seen when all test animals were treated with testosterone—possibly due to an increased sociability in the female group [17]. Since there are several differences between their study and ours with respect to study designs, type of paradigms and steroid treatment regimes in the other studies, strict comparisons of our results are problematic [17].

The results from the social discrimination paradigm propose that intact and GDX males, as well as intact females, display social recognition. The ability to discriminate between novel and familiar conspecifics seems however to be weaker in GDX females, which support the notion that estrogen facilitates social recognition in females [19, 28]. Since this study did not include information about estrus cycle phase we could not confirm its previously reported importance for social recognition [28, 29]. A number of investigations have shown that estrogen receptor-alpha, and to some extent estrogen receptor-beta, are crucial for social memory in females [18, 29, 30]. In contrast, the estrogen receptors influence long-term, but not short-term, social recognition in male mice [29]. Future studies need to further investigate if long-term social memory differs between males and females and to what extent androgen receptors are involved in social recognition. Additionally, early studies suggested that female and castrated male rats hold social memories for longer time than intact males [21, 31]. These effects cannot be seen in the current study as we did not test for long-term social recognition.

Our results from the novel object recognition paradigm showed that all groups have an intact object memory, but also that intact males display an elevated investigatory behaviour towards novel objects compared to females. Previous studies have shown that only intact and GDX male rats substituted with testosterone display object memory, in contrast to GDX males treated with oil or estradiol [32]. The differences in results may be explained by our considerably shorter inter-trial delay (30 vs. 90 min). In line with our results, Ceccarelli et al. [33], reported that male rats show more persistence investigating the objects compared to female rats. Also, GDX female and male rats showed shorter duration of investigation compared to intact females and males. Taken together, previous findings support the results of our study showing that all groups have object recognition memory although females showed lower levels of general interest in novel objects.

Previous studies have shown increased locomotor activity in GDX female mice when given estrogen treatment [30, 34, 35], and suggested this effect to be mediated by ER-alpha [36]. Konhilas et al. [37], and Houle-Leroy et al. [38], also showed that female mice had enhanced performance when tested in a wheel-running paradigm, compared to male mice. Ceccarelli and co-workers [33], reported that GDX male and female rats had lower locomotor activity compared to intact rats. We can, in our study, confirm that intact females have significantly higher locomotor activity compared to the three other groups of animals, but also that GDX males display decreased locomotor activity compared to intact females. As mentioned, this is interesting in relation to the social test where females displayed lower investigatory behaviour toward other animals and novel objects.

Both intact males and females showed sociability, social memory and object memory in our study. However, females consistently showed lower interaction times towards novel conspecifics and objects compared to males throughout all tests. Previous human studies using the face recognition paradigm, propose that women are better than men at encoding information, rather than having better face recognition memory as such [39]. In addition, it has been shown that more brain areas are activated during the encoding phase in men than in women [40, 41]. Based on these findings it was suggested that women more efficiently recruit relevant brain regions for encoding memories. To the best of our knowledge no studies have investigated differences in memory encoding in male and female mice. However, studies in humans give us reason to think that also female mice may encode social information more efficiently than males, which could contribute to explaining our results.

In conclusion, our results suggest that male mice investigate conspecifics more than females, and that these differences seem to be dependent on circulating hormones released from the testis. Our data further indicates that male mice may have a generally increased explorative behaviour compared to females. As these results seem to contrast with what is seen in humans, they should be taken in consideration when using the three-chambered apparatus, and similar paradigms as animal models of social deficits in e.g. autism. Already some few studies using animal models of autism have considered sex and gonadal status in their design, and hence found them to be important for social interest [42, 43]. Furthermore our results indicate that similar behavioural paradigms should be evaluated in animals, such as the zebrafish, whose social interactions are predominately dependent on vision rather than limited to olfactory/pheromonal signalling [44, 45].
